# Quantifying Liver-Stomach Disharmony Pattern of Functional Dyspepsia Using Multidimensional Analysis Methods

**DOI:** 10.1155/2020/2562080

**Published:** 2020-10-10

**Authors:** Zhongyu Huang, Zipan Lyu, Zhengkun Hou, Yuefeng Wu, Junqing Huang, Fengbin Liu, Jiaxu Chen

**Affiliations:** ^1^Integrated Chinese and Western Medicine Postdoctoral Research Station, Jinan University, Guangzhou 510632, China; ^2^The First Affiliated Hospital of Guangzhou Medical University, University of Chinese Medicine, Guangzhou 510632, China; ^3^Formula-Pattern Research Center, School of Traditional Chinese Medicine, Jinan University, Guangzhou 510632, China

## Abstract

**Purpose:**

This study aims to develop and validate a quantitative model for measuring severity of a typical traditional Chinese medicine (TCM) pattern for functional dyspepsia (FD) using multidimensional analysis methods including confirmatory factor analysis (CFA) and multidimensional item response theory (MIRT).

**Methods:**

A scale and theoretical models were constructed according to the definition of pathogenesis about “liver-stomach disharmony” patterns of FD. With data collected from 502 patients in a cross-section study, the theoretical model was validated with CFA, and the related validity and reliability were evaluated in Amos 21.0. By the use of the MIRT paradigm, psychometric properties of the scale were estimated and evaluated for pattern quantification.

**Results:**

A scale consisting of 12 items was constructed detecting three latent traits of the pattern. The theoretical model was evaluated to be with adequate consistency with clinical data as RMSEA < 0.05, CFI = 0.94, and *χ*^2^/D*f* = 2.29. As the correlation between symptoms and related pattern factors evaluated to be with adequate factor loading, the instrument is of preliminary interpretation. Most precision of assessment could be achieved for patients with moderate severity of the pattern as shown in test information and standard error functions.

**Conclusions:**

An instrument with an interpretable conceptual framework was developed for pattern quantification in TCM clinical practice. By constructing and evaluating both psychological and physical effects in a multidimensional model of the TCM pattern of FD, the paradigm raised in this article provided a valuable reference for interpreting complex diseases and theories such as FD and TCM patterns.

## 1. Background

Functional dyspepsia (FD) is one of the most commonly seen functional gastrointestinal disorders (FGIDs) that impact seriously on quality of life for many patients. The interplay of the psychological and physical effects of the disease was generally concerned, which leads to deterioration in the quality of life of individuals [1]. According to the definition of Rome IV, FD could be diagnosed with the presence of one or more of the following symptoms: epigastric pain or burning, early satiety, and postprandial fullness among which none of the structural disease using imaging or endoscopy could be observed [2]. Without practical objective targets for diagnosis or prognosis, curative effect of FD is unsatisfactory since diagnosis relied on complex clinical appearances always leading to confusion. It is of great necessity for physicians to have a strong foundation of knowledge and experience about treatment strategies for patients so as to identify and deal with the major cause and pathogenesis among the complex clinical appearances [3]. Talley also proposed the traditional paradigm of management of FGIDs is to be shifted to a more personalized and pathology-based model in place of symptom clustering [3]. However, a comprehensive evaluation of FGIDs is of difficulty since standardized quantitative detection approaches are still lacking. What's more, the overlapping of symptoms between different subtypes of FGIDs together with the influence of psychosocial, environmental, and dietary factors also makes it a challenge for disease management.

As an alternative and complementary approach, comprehensive treatment of TCM was reported to be with significant efficacy in releasing functional gastrointestinal disorders related symptoms [4]. Approaches such as TCM herbs and acupuncture had been reported as an effective alternative therapy in relieving related discomforts caused by FD through reducing visceral hypersensitivity and influencing central functional connectivity [5–7]. As a typical pattern of FD, the liver-stomach disharmony pattern reflects the relationship between emotional and digestive disorders that summarized as the correlated pathogenesis of “liver qi stagnation,” “spleen qi deficiency,” and “stomach qi counterflow” in TCM theory. With attempts to make rebalancing of individual status, it is one of the characteristics of TCM to address the importance of physical and mental regulation [4].

Although positive effects were observed, high-quality evidence of TCM research was rarely achieved out of empirical diagnosis in clinical practice. In TCM theory, pattern differentiation is an important procedure for the achievement of clinical efficacy by drawing a conclusion of disease pathogenesis drawn from clinical symptoms as a clinical diagnosis. However, there is a lack of objective evidence for the pattern theory. Fundamental concepts of TCM theory such as qi and zang-fu that derived from Chinese philosophy have yet to be mapped by scientific equivalents or elucidated in scientific terms [8]. The innovation in research methodology and application needs urgently to develop to promote the modernization of TCM based on abstract theory.

The concept of systematic management and individualized treatment of FD in modern medicine is also consistent with that in TCM. Due to the theoretical abstract of TCM, it is important to clarify the theoretical construction of pathogenesis to make a proper evaluation of patterns according to the severity of symptoms. Therefore, for the promotion of FD treatment with TCM treatment, it is of great necessity for the development of quantitative instruments based on adequate interpretation characteristics to assist in the comprehensive evaluation of FD patients.

As supplementary to the clinical evaluation, several instruments had been developed for individual condition measurement using both classical test theory and modern test theory, including structural equation modeling (SEM) [9], multidimensional item response theory (MIRT) [10] and computer adaptive tests (CAT) [11]. Instruments with quantitative characteristics such as GSRS [12] and FDDQOL [13] have been developed and widely used for evaluating severity and quality of life for FD patients. However, these scales could not meet the requirement of pattern evaluation of FD since they were not developed based on TCM theory.

In a previous study, a research paradigm was proposed for quantifying typical TCM patterns of diarrhea-predominant irritable bowel syndromes by the use of SEM and MIRT [14]. Latent variable analysis methods were evaluated to help quantify TCM patterns with a nonlinear and multidimensional relationship. Following the paradigm, this article attempts to quantify the typical TCM pattern of FD through the development and validation of a scale with multidimensional quantification characteristics for clinical assessment so as to promote the interpretation of the underlying architecture of TCM theory and clinical practice.

## 2. Methods

### 2.1. Data Source

A cross-sectional study was carried out in the First Affiliated Hospital of the Guangzhou University of Chinese Medicine between January 2017 and March 2018. Patients aged from 18 to 80 with a previous diagnosis of FD were assessed with a self-administrated scale by which typical symptoms of FD and related influencing factors were collected. All enrolled patients were diagnosed by senior clinical experts referring to the Roman IV criteria about FD [2, 15].

Following a description of the Consensus on diagnosis and treatment of functional dyspepsia with integrated traditional Chinese and western medicine [16], the liver-stomach disharmony pattern could be diagnosed with the presence of at least two primary symptoms or one secondary symptom as follows: Primary symptoms: gastric distention and fullness, ribs discomfort, and symptoms aggravated by emotion. Secondary symptoms: belching, sighs frequently, and edgy and irritable.

Patients meeting the diagnosis criteria were enrolled with informed consent. Any patient who does not meet the inclusion criteria or those who did not sign the informed consent form were excluded. Any case with an incomplete assessment with the percentage of absence or omitting answers of the self-administered items beyond 5% was also excluded.

### 2.2. Tool and Measurement

In this study, a scale was designed and developed according to the definition of liver-stomach disharmony pattern of FD in Internal Medicine of Chinese Medicine and the clinical guidelines for TCM clinical diagnosis and treatment of FD published by the Branch of Spleen and Stomach diseases of Chinese Society of Traditional Chinese Medicine [17]. Typical symptoms including “bloating,” “gastric distending pain,” “belching,” “early satiety,” “irritating,” “sigh,” and “stool inconsistency” as major clues for the diagnosis of the liver-stomach disharmony pattern of FD were drafted to build the theoretical model. And relieving and aggravating factors such as emotion and diet are included as additional information for the pattern analysis. Besides, signs of tongue and piles were not included in the theoretical model since there are no standard or quantitative criteria or tools for the collection.

As a cultural adjustment process of scale setting, the content of items as a description of the typical symptoms was evaluated under the supervision of 3 senior TCM experts. And options were designed as incremental degrees with scores 0 to 3 reflecting the severity of the symptoms, which was quantified according to the frequency and degree in the clinical practice following a suggestion from ROMA IV criteria. For severity measurement, 0 indicates none symptom and 1 indicates mild degree by which symptoms are not quite apparent and attack once a week. For a moderate degree, symptoms are apparent but do not disrupt daily activities, which occur 2–3 days a week. As to severe degree with score 3, symptoms are apparent and strongly affect daily life, which occurs 4–5 days or more in a week.

As shown in Table 1, the scale with 12 items was used for clinical assessment of liver-stomach disharmony pattern. For each patient diagnosed with FD, a trained researcher as a clinical practitioner would record the symptom severity and two clinical experts with senior titles were invited to evaluate the patient to his face to strengthen the accuracy of pattern diagnosis. Data collected in the assessment were then analyzed using a mixture of methods of latent variable analysis and classical statistical analysis.

**Figure 1:**
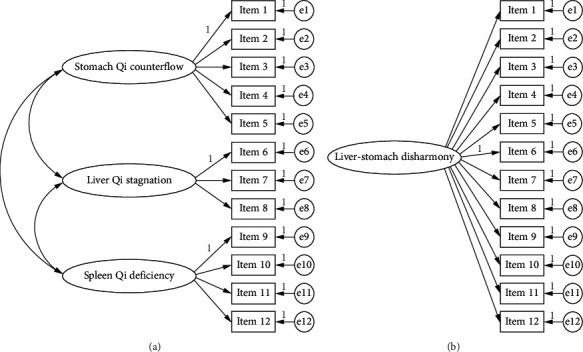
Multidimensional and unidimensional models for the liver-stomach disharmony pattern.

For extraction and quantification of the liver-stomach disharmony pattern, the theoretical model was disassembled according to the pathogenesis definition of the pattern. Three pattern factors including “stomach qi counterflow,” “liver qi stagnation,” and “spleen qi deficiency” were extracted and each item was attached to related factors following pattern differentiating logic and advice from TCM experts. The multidimensional theoretical model consisted of three pattern factors that were finally set up as shown in Figure 1 with the origin unidimensional model as a reference. Unidimensionality of the item setting was ensured without setting any symptoms or signs related to different pattern factors in the model.

### 2.3. Statistical Methods

The demographic characteristics of the enrolled patients were analyzed with descriptive statistics approach in SPSS 22.0. Cronbach's alpha coefficient was calculated to evaluate the validity of the scale with 0.60 as the lowest acceptable criteria. Psychometric properties including content validity, reliability, and construct validity were analyzed with confirmatory factor analysis (CFA) and MIRT.

According to the conceptual framework of the pattern and related symptoms, content validity was evaluated to test whether the instrument measures the typical pattern of FD adequately and sufficiently. The internal consistency of the items about each dimension of the model was measured with composite reliability indices. As the result of CFA, the construct validity of the instrument was evaluated with indices indicating goodness of fit between the conceptual framework and clinical data assessed such as comparative fit indices (CFI) and the root mean square error of approximation (RMSEA). The maximum likelihood method was used for estimating the model parameters including correlations and standardized regression weights among the exogenous variables in Amos 21.0. Moreover, construct reliability (CR) together with average variance extracted (AVE) were also estimated for measuring the construct reliability and convergent validity of the scale. Following suggestion from related researchers [18–21], indices and criteria of model properties evaluation of CFA are shown in Table 2.

Full-information item factor analysis of the multidimensional model of the liver-stomach pattern was performed with assist of packages including mirt [22] and mirtCAT [23] in *R* statistical software, version 3.4.2. Fitting item design of the scale, graded response model with the Quasi-Monte Carlo EM algorithm was used for the estimation of the three-dimension model following the methodology introduced in a previous study [14].

Coefficients of items under item response theory paradigm were estimated, and the multidimensional discrimination index (MDISC) was calculated as critical indices to show the overall measurement of each item about the capability in distinguishing between individuals [24]. An item is considered as high quality with its MDISC higher than 1, while an item is considered as moderate quality with its MDISC between 0.5 and 1.

Multidimensional difficulty index (MDIFF) [24] was also estimated to show the capability of each item in the fitting ability of individuals in the related factors. The information surface of both item level and test level was plotted in *R* statistical software as an intuitive display of psychometric characteristics of the scale.

## 3. Results

The demographics and characteristics of the 502 patients are shown in Table 3. A total number of 532 patients were diagnosed as FD by both Rome IV criteria and TCM criteria. There were 30 patients excluded in the study among which 8 did not fill the questionnaire while the others rejected to participate in the project. The median age of them was 37.19 years old with the youth took the greatest part (63.35%) among the sample while the old taking the least (1.99%). And the proportion of female patients is higher than that of males, which is consistent with previous reports about the prevalence of FD in China [25].

Internal consistency reliability was evaluated to be adequate with Cronbach's alpha coefficient of the whole items of the scale as 0.68. As shown in Table 4, the multidimensional model was evaluated to be with better construct reliability than that of the unidimensional model regarding the goodness of fit indices of both models. It was reported that the multidimensional model held better fitness indices with RMSEA < 0.05 and CFI = 0.94, while RMSEA and CFI about the unidimensional model were 0.15 and 0.42 and the Akaike information criterion (AIC) was reduced as the multidimensional model estimated. The multidimensional model showed adequate consistency with the clinical property of FD while the hypothesis of unidimensional setting was rejected.

As per the multidimensional model, parameters of the modeling fitting of each item were also estimated through CFA as shown in Table 5. It could be seen that most of the items, except item 3, item 5, and item 12, were evaluated to be with adequate factor loading to its theoretical related factor with standardized factor loading indices over 0.4. Composite reliability of each factor of the multidimensional model was evaluated to be adequate with indices of each factor over 0.6. Moreover, as could be seen in Table 6, three factors of the scale were with adequate discrimination ability as correlation indices between each factor were smaller than that compared with the factor themselves.

Full-information item factor analysis of the multidimensional model converged within 500 EM iterations. All items had at least an acceptable discrimination power (MDISC > 0.5), and some have a very high discrimination power with MDISC over 2.0 as shown in Table 7. Item-category parameters showed a descending trend as categories increased showing consistency with the theoretical setting of items.

**Figure 2:**
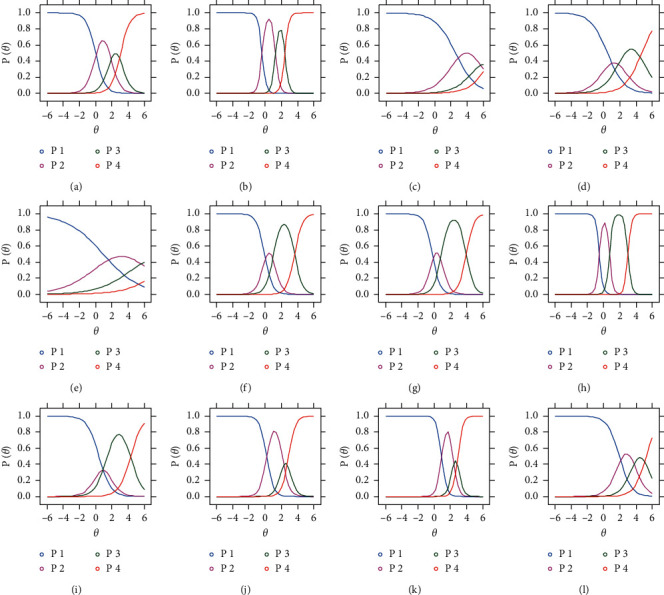
Trace lines for (a) item 1, (b) item 2, (c) item 3, (d) 4, (e) item 5, (f) item 6, (g) item 7, (h) item 8, (i) item 9, (j) item 10, (k) item 11, and (l) item 12.

Item trace lines of the scale were plotted as an intuitive display of psychometric characteristics of each item. As shown in Figure 2, most items showed adequate discrimination validity with the peak of curves distributing monotonously and orderly along with the theta value. For item 3 and item 5, peaks of curves of “moderate” to “severe” category were covered and overlapped by that of the “mild” category, indicating little information was added for patients at any level of severity. Options settings of the items need to be modified to fit and discriminate different levels of severity of FD patients.

**Figure 3:**
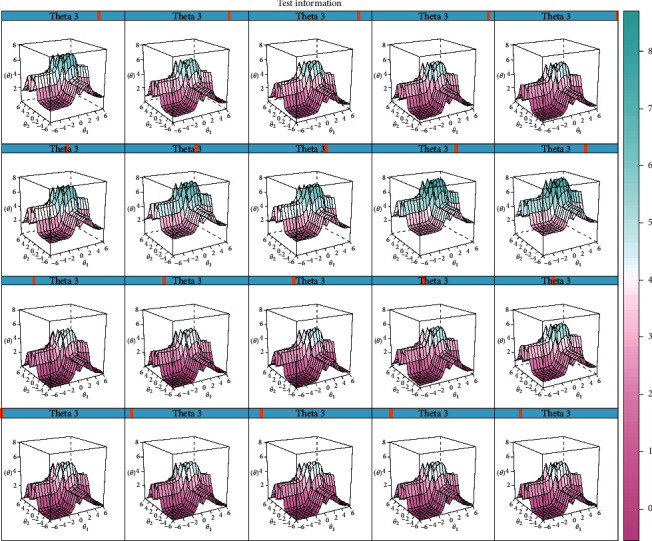
Test information surface of the scale for the multidimensional assessment.

**Figure 4:**
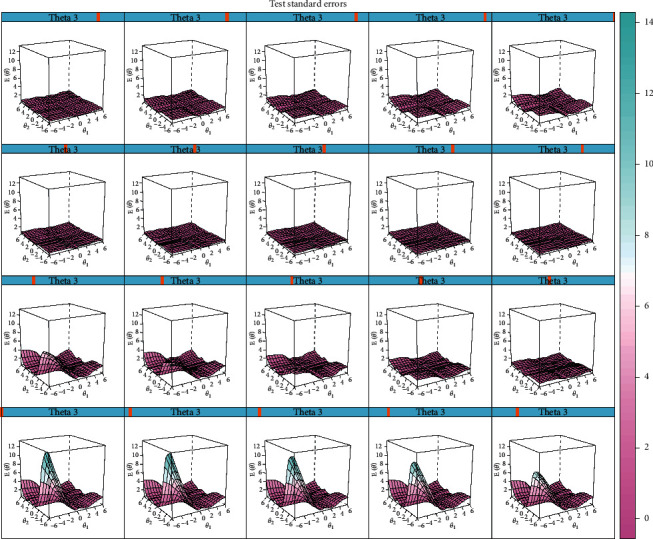
Test standard errors surface of the scale for the multidimensional assessment.

Test information surface and test standard error surface are plotted as shown in Figures 3 and 4. As to test information surface, it could be observed that the hump of surfaces appeared with all the three theta values in a range of (−2, 2), while a minimum of standard errors was achieved as shown in the test standard error surfaces. For individuals with moderate severity of the three latent traits, the best precision could be achieved through assessment with this instrument. Therefore, FD patients with moderate severity of “liver qi stagnation,” “spleen qi deficiency,” and “stomach qi counterflow” could be well differentiated using this instrument. However, for those with light severity, it may not be discriminative since there was the least information and large test standard error of the assessment with the estimated score of each factor in the range of (−6, −2).

## 4. Discussion

From the perspective of TCM, correlation and interaction between psychological factors and digestive symptoms of FD were evaluated with the 12-item scale designed for severity assessment of the liver-stomach disharmony pattern with a multidimensional theoretical model. With data collected from 502 patients, the reliability of the scale was evaluated to be at an acceptable level with Cronbach's alpha coefficients evaluated to be 0.68. The low internal consistency of the whole scale was mainly because of the scale design of multidimensional assessment properties. Meanwhile, the composite validity of each dimension of the scale was adequate with correlation variance between every two dimensions below the square root of AVE value of each dimension that is shown in the discrimination table. Neither test-retest nor the alternate-form reliability was evaluated due to the difficulty in performing for the state-related and dynamic character of TCM patterns.

Options setting of items in the scale were first set up according to suggestions from ROMA IV. Considering possible missing data in extreme conditions, the category of extreme serious was combined with the serious category to satisfy the requirement of MIRT analysis. Discrimination properties of most items and dimensions were evaluated to be adequate by CFA and MIRT. However, as shown in the ICC curves, symptoms such as belching in the severe level were rarely satisfied, indicating the setting of the scale was to be modified and revalidated in further practice. Moreover, the multidimensional theoretical model underlying the scale was evaluated with better fitness indices with clinical data than the unidimensional one, indicating the multifactor influence of FD. The test information and test error surfaces showed that the scale was most discriminative among FD patients with moderate severity of the pattern. The theoretical model was evaluated to be adequate, and precision indices about the test showed that the instrument was suitable for pattern traits evaluation for FD patients with moderate severity.

Estimation of statistical relation between symptoms and pathogenesis factors in the multidimensional model indicates an adequate representation of clinical recognition of the liver-stomach disharmony pattern of FD. And significant factor loadings of the relationships were estimated between gastric discomfort or dietary factor-related symptoms and the factor “stomach qi counterflow,” between emotional disorder-related symptoms and the factor “liver qi stagnation,” and between fecal abnormal symptoms and the “spleen qi deficiency.” It also offered adequate interpretation for the assessment practice for the comprehensive evaluation of emotional factors, bowel syndromes, and gastric syndromes, which was evaluated to be of high consistency with the theoretical definition of the liver-stomach disharmony pattern of FD.

The psychological property of FD has drawn great attention from researchers in the past decades [26, 27]. However, pathogenesis on how psychological factors affect FD patients is still unknown [28]. With an empirical theory, the correlation between psychology and physiology was put forward in ancient classic works of TCM. The theoretical definition of liver-stomach disharmony pattern reflects systematic knowledge of correlation between emotion and digestive activity of which a disorder of qi activity and its influence on conclusive organs including liver, spleen, and stomach were regarded as a hub for the pathogenesis. The pathogenesis of “liver qi stagnation,” “spleen qi deficiency,” and “stomach qi counterflow” are three of the major pathogenesis of digestion disorders in TCM theory. Liver stagnation could be caused by a mental disorder such as depression and anxiety through influencing the dispersion of qi, which further leads to an abnormality of the qi activity of spleen and stomach that is defined as qi deficiency and qi counterflow in TCM theory. As the stomach was defined as the recipient of food with qi fall as its normal activity, discomforts such as gastric distention and bloating occurred when there is a disorder of qi activity in the stomach that leads to the slower speed of digestion. The spleen was defined as the transporter of nutrients of the body; therefore, change of stool character and bodily discomforts such as fatigue could occur when there is a disorder that exists for the normal activity of transforming and transporting promoted by qi. However, the theoretical definition of the TCM pattern is indeed abstract and empirical. By clustering symptoms into factor-related groups according to the conclusive character of theoretical organ and activity, the model provided a systematic and more interpretable way for presenting the abstract theoretical logic of liver-stomach disharmony pattern. Moreover, in a quantifying framework, multidimensional analysis methods offered practical approaches for expanding knowledge of FD by integrating empirical theory and quantitative measurement.

This article showed a practical approach for the development and evaluation of a scale for the multidimensional assessment and with the expectation of providing interpretable appliances for treatment promotion of FD. However, there are still many limitations to overcome in this study. Firstly, only one pattern of FD was evaluated for the shortcoming of the single-center study design, which leads to an imbalance of data distribution. Further study with an expanded sample size of patients covering more patterns should be carried out to make a more practical scale for pattern differentiation as practical tools for assisting TCM clinical practice. As to the scale, more items covering related symptoms should be added to make comprehensive evaluation of FD patients especially for those with overlapping symptoms of functional gastrointestinal disorder. Secondly, although the sample size of this article was adequate, the scale and the underlying multidimensional model should be revalidated in further research and practice to achieve a more stable and convincing assessment result. Thirdly, the description of some item contents and the setting of options should also be revised and modified for better applicability and discrimination so as to reduce misunderstandings and avoid misleading for examinees towards the definition of the item. Last but not the least, interpretation of assessment results should be built to put forward clinical validation and optimization of the scale.

Regardless, this study provides a new instrument for quantifying the TCM pattern of FD underlying a multidimensional theoretical model that reflecting a systematic understanding of disease, a concept of individual diagnosis and treatment of TCM. By constructing and evaluating a multidimensional model of the TCM pattern of FD, the paradigm for quantifying a theoretical model with multidimensional analysis methods in this article provided a valuable reference for interpreting complex disease and theory such as FD and TCM patterns.

## 5. Conclusion

In this study, a scale with 12 items was designed for quantifying the severity of the liver-stomach disharmony pattern for FD patients. With adequate validity and reliability, the scale could be further applied for quantifying different traits of patients for a comprehensive measurement of FD. Multidimensional analysis methods including CFA and MIRT could be helpful for interpretation of complex theories such as TCM pathogenesis as a theoretical relation between symptoms and pathogenesis factors could be evaluated statistically. Further research should be carried out for modifying and revalidating the scale to promote knowledge of FD from the TCM perspective.

## Figures and Tables

**Table 1:** 12-item scale for assessment of liver-stomach disharmony pattern of FD.

Content of items	Definition of item categories			
	0	1	2	3
1 Postprandial epigastric distention	None	Mild	Moderate	Severe
2 Epigastric distending pain	None	Mild	Moderate	Severe
3 Beltching frequently	None	Mild	Moderate	Severe
4 Early satiety while eating	None	Mild	Moderate	Severe
5 Discomfort aggravated after meal	None	Mild	Moderate	Severe
6 Emotion-induced aggravation	None	Mild	Moderate	Severe
7 Edgy and irritable	None	Mild	Moderate	Severe
8 Feeling depressive	None	Mild	Moderate	Severe
9 Loose stool	None	Mild	Moderate	Severe
10 Sticky stool	None	Mild	Moderate	Severe
11 Fatigue	None	Mild	Moderate	Severe
12 Discomfort relieved after massage	None	Mild	Moderate	Severe

**Table 2:** Adequate criteria of indices of CFA for model properties evaluation.

Indices	Criteria	Related property
CFI	>0.9	Adequate goodness of fit
RMSEA	<0.05	Adequate goodness of fit
*χ* ^2^/df	<3	Adequate goodness of fit
Standardized factor loading	>0.4	Adequate goodness of fit
CR	>0.6	Adequate construct reliability
AVE	>0.25	Adequate convergent validity

CFI: comparative fit index; RMSEA: root mean square error of approximation; *χ*^2^: chi-square; df: degree of freedom.

**Table 3:** Demographics and characteristics of the 502 enrolled FD patients.

Variables	Total (*n* = 502)	Percentage
*Gender*		
Male	215	42.83
Female	287	57.17

*Age*		
Youth (18–40)	318	63.35
Middle age (41–65)	174	34.66
Old age (>65)	10	1.99

FD: functional dyspepsia.

**Table 4:** Indices for FD liver-stomach disharmony pattern models.

Indices	Unidimensional model	Multidimensional model
CFI	0.42	0.94
RMSEA	0.15	<0.05
AIC	699.34	171.24
*χ* ^2^	651.34	117.24
df	54	51
*χ* ^2^/df	12.08	2.29

FD: functional dyspepsia; CFI: comparative fit index; RMSEA: root mean square error of approximation; AIC: Akaike Information Criterion; *χ*^2^: chi-square; df: degree of freedom; *∗∗p* < 0.01.

**Table 5:** Estimates for the multidimensional model of liver-stomach disharmony pattern of FD.

Factor	Item	Unstd.	Std	SMC	CR	AVE
F1	Item 1	3.52^*∗∗∗*^	0.58	0.34	0.61	0.28
	Item 2	5.09^*∗∗∗*^	0.87	0.76	—	—
	Item 3	1.00^*∗∗∗*^	0.28	0.08	—	—
	Item 4	2.69^*∗∗∗*^	0.41	0.17	—	—
	Item 5	0.98^*∗∗∗*^	0.23	0.05	—	—

F2	Item 6	0.88^*∗∗∗*^	0.69	0.48	0.66	0.34
	Item 7	0.90^*∗∗∗*^	0.70	0.48	—	—
	Item 8	1.00^*∗∗∗*^	0.83	0.70	—	—

F3	Item 9	1.29^*∗∗∗*^	0.51	0.26	0.79	0.55
	Item 10	1.42^*∗∗∗*^	0.73	0.53	—	—
	Item 11	1.00^*∗∗∗*^	0.67	0.45	—	—
	Item 12	0.45^*∗∗∗*^	0.36	0.13	—	—

FD: functional dyspepsia; Unstd: unstandardized factor loading; Std: standardized factor loading; SMC: square multiple correlation; CR: composite reliability; AVE: average variance extracted; ^*∗∗∗*^*p* < 0.01.

**Table 6:** Factor discrimination indices of the multidimensional model of liver-stomach disharmony pattern.

Factor	AVE	F1	F2	F3
F1	0.28	0.53	—	—
F2	0.34	−0.09	0.58	—
F3	0.55	0.11	0.08	0.74

AVE: average variance extracted; F1: stomach qi counterflow; F2: liver qi stagnation; F3: spleen qi deficiency.

**Table 7:** Estimation of MIRT parameters of the items of the scale.

Items	MIRT parameters							
	a1	a2	a3	d1	d2	d3	MDISC	MDIFF
Item 1	1.73	0.00	0.00	0.00	−3.15	−5.33	1.73	0.00
Item 2	3.88	0.00	0.00	1.45	−5.02	−9.46	3.88	−0.37
Item 3	0.81	0.00	0.00	−2.08	−4.28	−5.82	0.81	2.58
Item 4	0.96	0.00	0.00	−0.47	−2.07	−4.52	0.96	0.48
Item 5	0.45	0.00	0.00	−0.41	−2.45	−4.35	0.45	0.91
Item 6	0.00	2.06	0.00	0.14	−2.14	−7.50	2.06	−0.07
Item 7	0.00	2.10	0.00	0.47	−1.84	−8.30	2.10	−0.23
Item 8	0.00	4.52	0.00	2.37	−3.29	−13.60	4.52	−0.52
Item 9	0.00	0.00	1.38	−0.56	−1.92	−5.98	1.38	0.41
Item 10	0.00	0.00	2.33	−0.45	−4.96	−6.75	2.33	0.20
Item 11	0.00	0.00	3.13	−2.84	−7.27	−9.18	3.13	0.91
Item 12	0.00	0.00	1.35	−2.62	−4.99	−7.09	1.35	1.94

MIRT: multidimensional item response theory; ai: discrimination parameter of dimension *i;* d*j*: easiness parameter of category *j* of an item; MDISC: multidimensional discrimination index; MDIFF: multidimensional difficulty index.

## Data Availability

The data used to support the findings of this study are available from the first author (huangzhongy@jnu.edu.cn) upon request.

## Abbreviations

TCM: Traditional Chinese Medicine
FD: Functional dyspepsia
CFA: Confirmatory factor analysis
MIRT: Multidimensional item response theory
FGIDs: Functional gastrointestinal disorders
SEM: Structural equation modeling
CAT: Computer-adaptive tests
CFI: Comparative fit indices
RMSEA: Root mean square error of approximation
CR: Construct reliability
AVE: Average variance extracted
AIC: Akaike information criterion
*χ*^2^: Chi-square
df: Degree of freedom
MDISC: Multidimensional discrimination index
MDIFF: Multidimensional difficulty index.
